# Occupational Health Surveillance Perspectives on e-SUS Linha da Vida Program

**DOI:** 10.1590/S2237-962220230004000014.en

**Published:** 2023-12-18

**Authors:** Cristiano Barreto de Miranda, Klauss Kleydmann Sabino Garcia

**Affiliations:** 1 Ministério da Saúde, Coordenação-Geral de Vigilância em Saúde do Trabalhador, Brasília, DF, Brazil

Occupational Health Surveillance (*Vigilância em Saúde do Trabalhador* - VISAT) encompasses actions aimed at promoting health, preventing morbidity and mortality, and reducing risks and vulnerabilities among Brazilian workers, regardless of their employment relationship, whether formal or informal.^1^ VISAT is consolidated through integrated actions that intervene in the determining factors of health problems arising from developmental models, production and work processes.^1^

Among its various responsibilities, VISAT conducts surveillance, prevention and control of workrelated diseases and injuries, aiming to promote healthy work environments and protect workers’ health.^2^ The high incidence of these diseases and health conditions, along with their impact on population morbidity and mortality, constitutes a serious public health problem, accounting for approximately 2 million global deaths annually, representing around 4% of the world’s gross domestic product (GDP).^3^ In Brazil, every 51 seconds, an occupational accident is registered, and approximately every 4 hours, a work-related fatality occurs.^4^ In addition to the human and social losses, the economic cost of these diseases and health conditions is equivalent to nearly BRL 350 billion (USD 714 million) for the country.^4^ These estimates are even more concerning because those accidents and diseases are preventable.

In the context of defining priorities and comprehensive care strategies, workers’ health information should be based on each territorial circumscription or specific regionalization. Thus, in November 2022, the Ministry of Health launched the e-SUS Linha da Vida Program with the objective of integrating national coverage notification systems, in order to gather health surveillance data on the entire Brazilian population.^5^ This program offers a promising perspective in improving the quality of data regarding work-related diseases and injuries, considering that the program aims to integrate available information on vital statistics and morbidities, and therefore, addressing the existing fragmentation of information on workers’ health among different Health Information Systems (HIS).

The objective of this article was to discuss the potential for improving occupational health surveillance actions, based on the consolidation of the e-SUS Linha da Vida Program.

## INTERFACES BETWEEN HEALTH INFORMATION SYSTEMS AND OCCUPATIONAL HEALTH SURVEILLANCE

It is worth noting that the HIS play a pivotal role in achieving VISAT’s goals. They help in a continuous and systematic manner in data collection, recording and monitoring work-related injuries, diseases and deaths. These systems also assist in recording exposures and health risk factors present in work environment and processes.^6^

In Brazil, the main HIS for VISAT encompass the Notifiable Health Conditions Information System (*Sistema de Informação de Agravos de Notificação* - SINAN), the Mortality Information System (*Sistema de Informações sobre Mortalidade* - SIM) and the Hospital Information System of the Brazilian National Health System (*Sistema de Informações Hospitalares do Sistema Único de Saúde* - SIH/SUS) (Box 1).^6^ Although the country has different data sources of interest to workers’ health, the lack of interoperability among them and the difficulty in accessing the identified data pose challenges to epidemiological monitoring and scientific research.

Given the lack of interoperability, an alternative approach that has gained prominence within VISAT, is the use of database linkage techniques, universally known as “record linkage”.^7^ However, the availability of probabilistic linkage methods and the accuracy of these techniques cannot avoid the direct influence of the quality of the user data, especially if the data has been input incorrectly.^7^ In addition, it is important to consider the absence of a unique and consistent identifier between the systems, such as the Taxpayer Identification number (*Cadastro de Pessoa Física* - CPF) or the National Health Card (*Cartão Nacional de Saúde* - CNS) number.

In this context, the e-SUS Linha da Vida Program stem from the necessity of having a single and reliable source of data. A need that was especially recognized during the COVID-19 pandemic.^5^

It is worth mentioning the substantial underreporting of work-related diseases and injuries, which poses a challenge to the effectiveness of VISAT’s actions,^8^ despite the recent increase in the number of reported cases on SINAN.^9^ Underreporting hinders a more comprehensive understanding of the underlying factors behind accidents and illnesses, making it difficult to implement targeted interventions.

Some limitations of the current HIS that are relevant to VISAT, include the absence of return flow – data sharing between the municipalities of notification and the case’s residence – for the notifications of work-related diseases and injuries, on SINAN.^10^ Furthermore, the coding lists for the “occupation” field, based on the Brazilian Classification of Occupations (*Classificação Brasileira de Ocupações* - CBO), and the “economic activity” field, based on the National Classification of Economic Activities (*Classificação Nacional de Atividades Econômicas* - CNAE), show that the HIS have not been updated. These lists do not include new occupation codes, which are created anually and introduced into the systems of the Ministry of the Labor and Employment of Brazil,^11^ nor do they include the codes from CNAE in its version 2.0, implemented in 2007.^12^ SINAN, specifically, uses CNAE version 1.0, 2002.^13^

Another limitation of the HIS lies in the absence of fields that enable the identification of the relationship between illness and work in some notification forms, such as the individual tuberculosis notification/investiogation form.^14^ On the other hand, in the SIM, the “occupational accident” field in the Death Certificate (DC) only allows registration for deaths due to external causes, specifically accidents (ICD-10: V01 to X59). This prevents work-related connection for deaths resulting from non-communicable diseases, communicable diseases, homicides, suicides and other forms of violence.^15^

The obstacles encountered in different HIS compromise the epidemiological investigation process of work-related cases and deaths, with a negative impact on data quality and, consequently, on worker’s health information. In light of the recent transformations in the world of work, boosted by the COVID-19 pandemic, epidemiological surveillance models for occupational health and HIS need to be reconsidered to equally address the social determinants of health, especially those related to work process and environment.

**Box 1:** Main variables of the Notifiable Health Conditions Information System (*Sistema de Informação de Agravos de Notificação* - SINAN), Mortality Information System (*Sistema de Informações sobre Mortalidade* - SIM) and Hospital Information System of the Brazilian National Health System (*Sistema de Informações Hospitalares do Sistema Único de Saúde* - SIH/SUS) of interest to occupational health surveillance

SINAN	SIM	SIH/SUS
**Worker characterization variables** Name Age Sex Race/skin color Schooling Pregnant employee Address information	**Worker characterization variables** Name Age Sex Race/skin color Schooling Address information	**Worker characterization variables** Name Date of birth Sex Race/skin color Address information
**Work-related variable** Occupation – CBO^a^ Economic activity – CNAE^b^ Labor market status Length of employment in the occupation Data on the contracting company Workplace accident notification	**Work-related variable** Deceased’s usual occupation – CBO^a^ Mother’s usual occupation, for fetal deaths and deaths in children under 1 year old – CBO^a^	**Work-related variable** Occupation – CBO^a^ Economic activity – CNAE^b^ Social Security beneficiary CNPJ^c^ of the contracting company
**Characteristic of the health condition/illness variables** Work-related disease Specific diagnosis (of the accident and injury) Other workers affected by the health condition/illness Type and time of the accident Time of exposure to the risk agent Treatment regimen General approach adopted Case progression	**Variables related to conditions and causes of death** Place of death Causes of death Type of circumstances of unnatural death Work-related accident Type and address of the place where the accident or violence occurred Municipality of occurrence	**Variables of conditions and causes of hospitalization** Initial diagnosis ICD-10^d^ - primary, secondary and associated codes Type of accident: traffic accident, typical work-related accident and commuting accident

a) CBO: *Classificação Brasileira de Ocupações* (Brazilian Classification of Occupations); b) CNAE: *Classificação Nacional de Atividades Econômicas* (National Classification of Economic Activities); c) CNPJ: *Cadastro Nacional da Pessoa Jurídica* (National Register of Legal Entities); d) ICD-10: International Statistical Classification of Diseases and Related Health Problems – 10^th^ Revision. Sources: SINAN Notification/Investigation forms, Death Certificate (DC) and Hospital Admission Authorization via the Brazilian National Health System (*Autorização de Internação Hospitalar pelo Sistema Único de Saúde* - AIH-SUS).

## VISAT PERSPECTIVES ON THE e-SUS LINHA DA VIDA PROGRAM

The e-SUS Linha da Vida Program aims to compile individual information – currently present in different HIS^16^ – from birth to death, gathering information on health conditions, diseases, hospitalizations, etc., within the system. This Program represents a significant change in the HIS approach as it focuses on the individual, rather than the disease or health condition. It is estimated that the e-SUS Linha da Vida Program will be implemented in the Brazilian health surveillance services by 2025.^5^

The person-centered focus provides a more comprehensive perspective on the relationship between health and work, enabling an understanding of different cycles and potential impacts of work on an individual’s health over time. This approach is crucial because it expands the detection of work-related diseases, given that many of them have a long latency period, making it challenging to immediately identify the causal relationship with current or previous work.^17^ By enabling the recording of occupational history and economic activity, the Program makes it possible to know the potential occupational risks to which an individual has been exposed throughout his or her life. This quality will enable a more accurate – and effective – identification of the workers’ health status, the prioritization of the most affected groups and the planning of more appropriate intervention actions.

The Program’s main potential is also due to data standardization and integration of different information sources. Based on a minimum data set (MDS) and the adoption of standardized terminologies, the e-SUS Linha da Vida Program aims to establish a common language and a consistent data structure.^7^ Data standardization will solve, for example, the problem of non-interoperability between the HIS, which has been addressed through record linkage techniques to date.^6^ Furthermore, standardization will enable the use of MDS in integrating with databases that do not belong to the health sector, such as the Occupational Accident Information System (*Sistema de Informação de Acidentes de Trabalho* - SISCAT), of the Ministry of Social Security, and the Annual Social Information Report (*Relação Anual de Informações Sociais* - RAIS), of the Ministry of Labor and Employment, databases that provide information on Brazilian workers.

## FINAL CONSIDERATIONS

Fast and safe access to critical information is essential for health decision-making and the timely implementation of prevention measures in the workplace, in addition to contributing to the identification of emerging problems related to workers’ health.

The e-SUS Linha da Vida Program creates opportunities for strengthening VISAT by enabling proper monitoring and greater understanding of the effects of work on an individual’s life. This, in turn, can lead to the improvement of the analysis of the occupational health situation and the development of surveillance policies and actions, reinforcing the National Network of Comprehensive Occupational Health Care (*Rede Nacional de Atenção Integral à Saúde do Trabalhador* - RENAST); and, consequently, expanding measures for the prevention of work-related diseases and injuries, offering the possibility of implementing a systemic and reliable monitoring of workers’ health in Brazil.
